# Developmental maturation of astrocytes and pathogenesis of neurodevelopmental disorders

**DOI:** 10.1186/1866-1955-5-22

**Published:** 2013-08-29

**Authors:** Yongjie Yang, Haruki Higashimori, Lydie Morel

**Affiliations:** 1 Department of Neuroscience, Tufts University School of Medicine, 136 Harrison Ave, Boston, MA 02111, USA; 2 Neuroscience Program, Tufts Sackler School of Graduate Biomedical Sciences, 136 Harrison Ave, Boston, MA 02111, USA

**Keywords:** Astrocyte, Developmental maturation, Neuronal to astrocyte signaling, Glutamate, Glutamate transporter, GLT1, Developmental disorder

## Abstract

Recent studies have implicated potentially significant roles for astrocytes in the pathogenesis of neurodevelopmental disorders. Astrocytes undergo a dramatic maturation process following early differentiation from which typical morphology and important functions are acquired. Despite significant progress in understanding their early differentiation, very little is known about how astrocytes become functionally mature. In addition, whether functional maturation of astrocytes is disrupted in neurodevelopmental disorders and the consequences of this disruption remains essentially unknown. In this review, we discuss our perspectives about how astrocyte developmental maturation is regulated, and how disruption of the astrocyte functional maturation process, especially alterations in their ability to regulate glutamate homeostasis, may alter synaptic physiology and contribute to the pathogenesis of neurodevelopmental disorders.

## Review

Neurodevelopmental disorders are a wide range of clinical conditions that impair growth and development of the central nervous system (CNS) during early postnatal stages. In particular, autism spectrum disorder (ASD), a class of conditions defined by deficits in social behavior and communication [1], represents a major category of neurodevelopmental disorders. Although the exact causes for these disorders remain largely unknown, earlier genome-wide association studies (GWAS) [2-4] to recent exosome sequencing [5-8] have associated over 1,000 genes with various forms of neurodevelopmental disorders [9], including ASD, implicating a strong genetic component in their etiology. In particular, genetic mutations of methyl CpG binding protein 2 (MeCP2) [10] and fragile X mental retardation 1 (fmr1) [11] genes have been identified to cause the monogenic neurodevelopmental disorders Rett syndrome and Fragile X syndrome (FXS), respectively. Interestingly, both Rett syndrome and FXS significantly resemble typical autistic phenotypes [12,13]. The studies on MeCP2/fragile X mental retardation protein (FMRP, protein product of fmr1 gene) function and how their mutations cause Rett syndrome and FXS therefore provide valuable clues to the pathogenesis of the sporadic forms of neurodevelopmental disorders. In addition to the identification of genetic risk factors, direct morphometric, neuropathological, and functional neuroimaging studies have characterized important pathological features in autistic brains, such as enlarged brain size (head circumference), decreased numbers of cerebellar purkinje neurons, increased inflammatory responses, and altered brain connectivity and activity (increased susceptibility to seizures) [14,15].

Although pathological features of autistic brains are mostly neurocentric, it has gradually become evident from recent studies that (astro)glial cells are likely to be active components in the pathogenesis of ASD and other neurodevelopmental disorders [16,17]. Astrocytes are known to significantly modulate synaptogenesis during development [18-20] and play diverse and active roles in synaptic physiology in the adult brain [21,22]. Despite the essential roles of astrocytes at functional synapses, how astrocytes are generated and become morphologically and functionally mature during development remains largely uncharacterized. It also remains unknown whether astrocyte development becomes impaired in neurodevelopmental disorders and how impairment in astrocyte development may contribute to pathological features observed in various neurodevelopmental disorders. In this review, we will summarize recent progress in understanding astrocyte development and their involvement in neurodevelopmental disorders. Although several recent excellent reviews have described the involvement of astrocytes in neurodevelopmental disorders [16,17], we will give our own perspectives about the morphological and functional maturation of astrocytes during development, how astrocyte developmental maturation is regulated, and how the disruption of the functional maturation process of astrocytes, especially alterations in their ability to regulate glutamate homeostasis, may alter synaptic physiology and contribute to the pathogenesis of neurodevelopmental disorders.

### Developmental maturation of astrocytes in the CNS

All of the CNS cell types except the microglia are differentiated from neural stem cells (NSC) at different stages of embryogenesis. It is well characterized that glial cells, including astrocytes are generated following neuronal differentiation from NSC during early embryogenesis. The peak of astrogliogenesis occurs late prenatal to early postnatal stages, that is, E18-P7 in various rodent CNS regions [23,24]. The developmental stages referred in this review are based on the mouse CNS development unless otherwise stated. Recent studies have found several pathways/mechanisms that are involved in astrocyte fate specification from NSC, including Janus kinase (JAK)/Signal Transducer and Activator of Transcription (STAT) [25], Bone morphogenetic protein (BMP)-SMAD [26,27], Notch [28], and Nuclear factor IA (NFIA) [29]. These pathways are activated by the extrinsic gliogenesis signals, including Ciliary neurotrophic factor (CNTF), Cardiotrophin 1 (CT*-*1) [30], and Leukemia inhibitory factor (LIF) [31], which are secreted from early differentiated neurons or late-stage NSCs. These signals also induce intrinsic changes, especially the epigenetic modifications to open the appropriate chromatin regions [31], which allows binding of transcriptional factors (STAT, SMAD, or NFIA) [32] to astroglial gene promoters (such as GFAP, S-100β, and GLAST) to induce their expression [27,29,33]. Although early *in vitro* studies suggest the presence of bipotent glial restricted cells (GRP) that can generate both oligodendrocytes and astrocytes [34], recent *in vivo* studies have shown that GRP is likely to be a minor pathway in gliogenesis [35]. The identity of astrocyte progenitor cells *in vivo* remains elusive. Interestingly, a recent study has shown that local proliferation of differentiated astrocytes is the major source of astroglia in the postnatal cortex [36]. Early produced astrocytes continue cell divisions while also undergo differentiation, implicating a progenitor status of early produced astrocytes [36].

**Figure 1:**
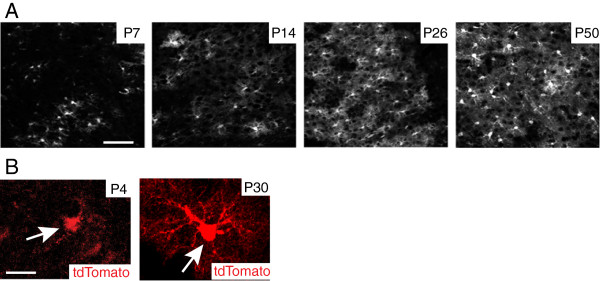
**Morphological maturation of astrocytes during postnatal development. (A)** Growth of cortical astrocyte processes illustrated with tdTomato reporter on EAAT2 tdTomato transgenic mice during postnatal development; Scale bar: 100 μm; **(B)** A magnified view of typical astrocyte morphology at P4 and P30 from cortex of EAAT2 tdTomato mice. Scale bar: 50 μm.

Adult astrocytes, regardless of their anatomical location (gray or white matter), typically occupy a large and non-overlapping domain, which is composed of a large number of branches and fine processes [37]. In particular, these fine processes (typically <50 nm in diameter) are typically distal from the soma and are generally GFAP immunostaining negative. These fine processes, also termed ‘peripheral astrocyte processes (PAPs)’ [38], constitute roughly 50% of the mature astrocyte volume and 80% of the surface area [39,40], permitting sufficient insertion of various membrane proteins such as ion channels, ligand receptors, and transporters. PAPs extensively contact synapses and are considered primary sites for active astrocyte and neuron signaling [41]. Although a significant number of astrocytes are largely generated during the first week postnatally [36,42], these fine processes (PAPs) are not induced in these astrocytes until several weeks later (Figure 1) [31,43]. The astroglial network, indicated by the interacting PAPs, is also formed at later developmental stages (P14 to P26, Figure 1). In addition to morphological maturation, several important astroglial genes, including glutamate transporter GLT1 (rodent analog of human excitatory amino acid transporter 2) [44,45], connexin 43 and 30 [46], and inwardly rectifying potassium channel Kir4.1 [47,48], are also induced in astrocytes within 3 to 4 weeks after birth, suggesting that astrocytes also undergo dramatic molecular changes during developmental maturation. Interestingly, these astroglial genes represent some of most characteristic and important functions of astrocytes in the CNS. For example, GLT1 is the physiologically dominant glutamate transporter in the CNS to clear extracellular glutamate; connexin 43 and 30 are the major components of the astrocyte gap-junction which forms the astrocyte network; Kir4.1 is an important potassium channel in astrocytes that is critical for maintaining the K^+^ gradient for proper glutamate uptake and also significantly buffers activity-induced K^+^ release. Induction of these genes during development, together with the growth of PAPs in astrocytes, suggests that astrocytes undergo a developmental maturation phase from the first week to the following 2 to 3 weeks postnatally to acquire their unique morphology and molecular function. Notably, this is a developmental stage that follows the early astrocyte fate specification and differentiation. Moreover, the molecular maturation is tightly associated with the morphological maturation of astrocytes, as these important proteins mentioned above are all membrane proteins that are primarily localized on the surface of the fine processes for their proper functions in astrocytes.

**Figure 2:**
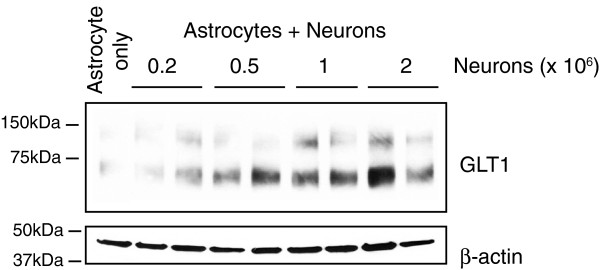
Expression of GLT1 in cultured astrocytes is induced by co-cultured neurons in a dose-dependent manner.

Despite the dramatic changes astrocytes undergo during the maturation phase, the mechanisms for astrocyte developmental maturation remain largely unknown. Neuronal signaling has been implicated in early astrocyte fate specification by secreting the extrinsic signals like CT-1 [30] from early-differentiated neurons to induce astrocyte differentiation. Interestingly, although GLT1 is the predominant glutamate transporter in adult brain, cultured astrocytes express minimal levels of GLT1 [49]. Instead, GLAST (rodent analog of human excitatory amino acid transporter 1, EAAT1) is the dominant glutamate transporter in cultured astrocytes [50]. However, when astrocytes are co-cultured with neurons, GLT1 expression in astrocytes is highly induced in a dose-dependent manner (Figure 2) [51,52]. Similarly, expression of connexin 43/30 is also induced in astrocytes when co-cultured with neurons [53,54]. As cultured astrocytes share a more similar gene profile with immature astrocytes *in vivo* (P1-P7) [55], neuron-dependent induction of these astroglial genes in cultured astrocytes indicates that neuronal signaling is likely to play important roles in induction of astrocyte gene expression during development *in vivo*. Recent gene expression profile studies in astrocytes that are acutely isolated from the brain have revealed that developing astrocytes express many neurotransmitter receptors [55,56], allowing them to receive neuronal signals. Indeed, a substantial body of literature has reported that neuronal activity triggers Ca^2+^ changes in astrocytes *in vitro* and *in vivo*[57,58], though the specific metabotropic glutamate receptor that mediates this response varies significantly between immature (GRM5) and mature astrocytes (GRM3) [59]. In addition, although it is unclear whether neuronal activity modulates PAPs formation in astrocytes during developmental maturation, direct application of glutamate induces rapid filopodia motility in cultured astrocytes [60]. Neuronal activity has also been closely associated with structural plasticity of adult astrocytes *in vivo*[61]. Both secreted neuronal signals, such as activity-dependent neurotransmitters and small protein CT-1 and contact-dependent signaling, such as Notch-JAG1 interaction [62], can play important roles in astrocyte developmental maturation. Molecular mechanisms for astrocyte maturation, especially downstream pathways that mediate potential neuronal signaling are essentially unknown. As various pathways/mechanisms involved in early astrocyte specification are characterized (described above), it would be interesting to test whether any of these pathways also regulate developmental maturation of astrocytes at a later stage.

### Roles of astrocytes in neurodevelopmental disorders

The first evidence of potential astrocyte abnormalities in neurodevelopmental disorders was from biochemical analysis of patient brain samples and screening of genetic risk factors for various forms of neurodevelopmental disorders. Astrogliosis, indicated by increased GFAP expression, was found in the cerebellar cortex of autistic brains [63], though neuronal degeneration was not generally observed in brains of neurodevelopmental disorder patients. Expression changes of a few other astroglial proteins were also observed in brain samples of ASD patients. For example, increased EAAT2 and EAAT1 expression was found in cerebellum of autistic patients [64]. Significantly increased connexin 43 was found in the superior frontal cortex, while decreased aquaporin 4 was found in the cerebellum of autistic brains [65]. These astroglial changes in autistic brains imply that astrocytes are likely to be involved in neurodevelopmental disorders. In addition, genetic studies have found associations between specific nucleotide polymorphisms in the EAAT1 sequence with severity of repetitive behaviors and anxiety in ASD children [66]. Mutations of Kir4.1 were also found in a subset of autism patients [67], which likely affects the K^+^ homeostasis in the brain and increases the seizure susceptibility in autism patients. Despite these clinical studies, it is important to note that specific astrocyte-related mechanisms involved in the pathogenesis of neurodevelopmental disorders remain to be characterized.

Significant progress in understanding the roles of astrocytes in neurodevelopmental disorders has recently been made from animal (mouse) models of two typical monogenic neurodevelopmental disorders: Rett syndrome and FXS. Loss-of-function mutations in MeCP2 are the primary cause for the vast majority of Rett syndrome patients [10]. The fragile X syndrome is caused by the transcriptional silencing of FMRP expression, as a result of hypermethylation on the abnormally high number (>200) of trinucleotide CCG insertions at the *fmr1* gene locus [11]. Both MeCP2^−/−^[68,69] and fmr1^−/−^[70] mice were later developed that recapitulate typical clinical symptoms of Rett syndrome or FXS, respectively. Extensive studies have been carried out in these mouse models to understand the functions of MeCP2 and FMRP in brain and how their loss-of-function mutations alter dendrite morphology and synaptic physiology, which underlies the clinical phenotypes of Rett syndrome and FXS [71,72]. Using these mouse models, recent studies have started to unveil the pathogenic roles of astrocytes in neurodevelopmental disorders. Astrocytes derived from MeCP2^−/−^ mice can significantly affect normal neuronal development [73]. Wild-type hippocampal neurons co-cultured with MeCP2-deficient astrocytes or treated with astrocyte conditioned medium (ACM) collected from MeCP2-deficient astrocytes exhibit abnormally stunted dendrites [73]. Similarly, astrocytes derived from fmr1^−/−−^ mice also induce developmental delays in dendrite maturation and synaptic protein expression of hippocampal neuronal dendrites in co-cultures [74,75]. Excessive neurotrophin-3 (NT-3), but not other growth factors secreted from fmr1^−/−^ astrocytes, was later suggested to reduce the dendrites of neurons and synaptic protein levels in FXS condition [76]. In addition, loss of MeCP2 in astrocytes appears to be dependent upon the astroglial gap-junction [77] and it induces expression changes of several important astroglial genes [78]. Most interestingly, selective restoration of MeCP2 in astrocytes *in vivo* using the Cre-loxP recombination system significantly improves locomotion and anxiety levels, and restores respiratory abnormalities to a normal pattern [79]. At the cellular level, re-expressed MeCP2 in astrocytes also restores normal dendritic morphology and increases levels of the vesicular glutamate transporter VGLUT1 [79]. These results clearly suggest that astrocytes play important pathogenic roles in Rett syndrome and they should also be considered as important therapeutic targets, in addition to neurons [16].

Glutamate homeostasis is essential for brain physiology. Proper glutamatergic signaling is important in regulating neurite outgrowth, synaptogenesis, neuronal migration, differentiation, and cell death in the developing brain [80-82]. Given its important and diverse functions in development, it is not surprising that altered glutamatergic signaling may significantly contribute to the pathogenesis of neurodevelopmental disorders. Indeed, several genetic studies have associated variations of glutamate receptor genes (GluR6, GRM8, and GRIN2A) with ASD [83-85]. In addition, studies using fmr1^−/−^ mice have characterized the abnormally increased group I mGluR (especially mGluR5) activation-induced protein synthesis [72,86], which underlies several typical abnormalities observed in FXS, such as enhanced mGluR-dependent long-term depression (LTD) [87], induction of elongated/immature dendrites [88], and increased susceptibility to audiogenic seizure [89], and so on. Although FMRP is generally considered a translation suppressor and increased mGluR1/5-dependent dendritic protein synthesis is largely due to the loss of FMRP in neurons in FXS [86], the observation that genetic or pharmacological inhibition of mGluR5 activation effectively and significantly reduces mGluR1/5-dependent dendritic protein synthesis in fmr1^−/−^ mice [90,91] also implicates that mGluR1/5 activation is abnormally enhanced in FXS. In addition, inhibition of mGluR5 signaling also significantly suppresses repetitive behaviors and social behavior deficits and reverses elevated stereotypical and anxiety-like behaviors in mouse models of idiopathic autism [92], suggesting that abnormally enhanced mGluR5 activation may also contribute to some forms of idiopathic autism. In the mammalian CNS, neuronal mGluR1/5 is preferentially localized on the peri-synaptic surface membrane, thus its activation is highly dependent upon the glutamate that is spilled out from the synaptic cleft [93]. Interestingly, the extracellular, especially the spilled glutamate levels are tightly regulated by astroglial glutamate transporters GLT1/GLAST (human EAAT2/EAAT1) [94]; therefore the activation of neuronal mGluR1/5 is actively modulated by astroglial GLT1/GLAST expression/activity. Indeed, pharmacological inhibition or genetic deletion of GLT1/GLAST activity potentiates postsynaptic neuronal mGluR activation [95], while upregulation of GLT1 expression severely impairs mGluR-dependent LTD at rat mossy fiber-CA3 synapses [96].

**Figure 3:**
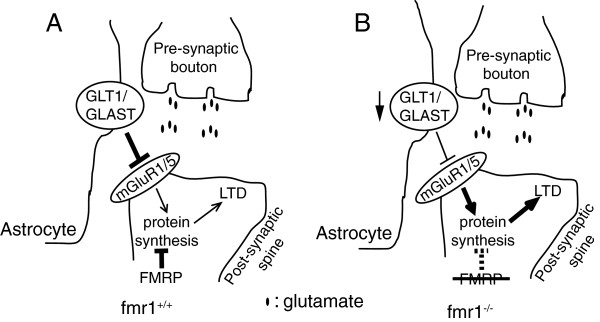
**Dysregulation of astroglial glutamate transporters is likely to enhance neuronal mGluR1/5 activation and further increases downstream protein synthesis in FXS condition.** Synaptic neuron to astrocyte communication in **(A)** fmr1^+/+^ and **(B)** fmr1^−/−^ conditions. LTD: Long-term depression.

We have recently found a downregulation of GLT1/GLAST expression and reduced glutamate uptake in cortex of fmr1^−/−^ mice during postnatal development and have shown that their downregulation contributes to the enhanced neuronal excitability observed in fmr1^−/−^ mouse cortex [97]. These results suggest that the dysregulated GLT1/GLAST expression is likely to be an upstream, astrocyte-mediated mechanism leading to enhanced neuronal mGluR5 activation in FXS. As a result, enhanced neuronal mGluR5 activation may synergistically increase abnormal dendritic protein synthesis, together with the loss of FMRP-mediated suppression on protein synthesis in fmr1^−/−^ neurons (Figure 3). It also explains the earlier observations that the basal levels of protein synthesis are elevated in fmr1^−/−^ mice [86]. In addition, proper expression of GLT1 and GLAST is known to be essential for the normal development of the CNS [81] and GLT1^−/−^ mice exhibit severe seizures as early as P14 [98]. Moreover, 30% of GLT1^+/−^ GLAST^+/−^ (double heterogeneous) mice with 50% of GLT1 and GLAST expression levels (and 30% increased extracellular glutamate levels [99]) exhibit behavioral and neuroanatomical abnormalities often observed in autism, including abnormal social interaction, seizures, and an enlarged amygdala and hippocampus [100]. As astroglial glutamate transporters, especially GLT1, are strongly induced during the functional maturation process, potential dysregulation of GLT1/GLAST during development exemplifies how alterations in the functional maturation of astrocytes could contribute to the pathogenesis of FXS and other neurodevelopmental disorders.

Astrocytes perform many other important functions in mammalian CNS, in addition to the clearance of extracellular glutamate. Developing but not mature astrocytes secrete thrombospondins 1 and 2 (TSP-1 and TSP-2), which significantly and specifically increases numbers of excitatory synapses [101]. Glypican 4 (Gpc4) and glypican 6 (Gpc6) were further identified from astrocyte conditioned medium to promote glutamate receptor clustering and receptivity, which facilitates the formation of postsynaptically functioning CNS synapses [18]. Additional synaptogenic molecules secreted from astrocytes are being characterized [102]. Although immature dendrite morphology and altered synaptic functions are hallmarks in many neurodevelopmental disorders, whether these developing astrocyte-dependent synaptogenic pathways are altered and their consequences in neurodevelopmental disorders remain to be examined. In addition, we have very limited knowledge about the molecular changes of astrocytes in neurodevelopmental disorders, especially from *in vivo* settings. Examination of molecular changes in astrocytes *in vivo* has been traditionally difficult; however, recent development of an array of astrocyte reporter mice (BAC GLT1 eGFP [103], BAC ALDH1L1 eGFP [55], EAAT2 tdTomato [104]) has allowed rapid isolation of *in vivo* astrocytes from mouse models of neurodevelopmental disorders through fluorescence activated cell sorting (FACS) and subsequent genome-wide transcriptional profiling. The development of translational ribosome affinity purification (TRAP) technique and ALDH1L1-TRAP mice also provides another convenient *in vivo* approach to isolate translating mRNAs from astrocytes in an unprecedented temporal and spatial manner [105,106]. These tools will greatly facilitate the molecular characterization of potential developmental abnormalities of astrocytes in various neurodevelopmental disorders. Results from these studies will provide valuable clues about the pathways that regulate functional maturation of astrocytes during development and their molecular alterations in neurodevelopmental disorders.

## Conclusions

In summary, we discuss the functional maturation of astrocyte during postnatal development, and the disruption of this maturation process, like the dysregulation of glutamate transporters may serve as an astrocyte-dependent mechanism for the pathogenesis of FXS and other neurodevelopmental disorders. Although the molecular mechanisms of astroglial maturation and how the disruption of this maturation process contributes to the pathogenesis of neurodevelopmental disorders remain to be investigated, we expect that the availability of novel *in vivo* tools for astrocyte study will greatly help answer these questions. Ultimately, a better understanding of the roles of (astro)glia in the pathogenesis of neurodevelopmental disorders will facilitate the search for cures for these disorders.

## Abbreviations

ASD: Autism spectrum disorder; BMP: Bone morphogenetic protein; CNS: Central nervous system; CNTF: Ciliary neurotrophic factor; CT-1: Cardiotrophin 1; EAAT: Human excitatory amino acid transporter; FACS: Fluorescence activated cell sorting; Fmr1: Fragile X mental retardation 1; FMRP: Fragile X mental retardation protein; FXS: Fragile X syndrome; GRM: Metabotropic glutamate receptor; GWAS: Genome-wide association studies; JAK: Janus kinase; Kir: Inwardly rectifying potassium channel; LIF: Leukemia inhibitory factor; LTD: Long-term depression; MeCP2: Methyl CpG binding protein 2; NFIA: Nuclear factor IA; NSC: Neural stem cells; NT-3: Neurotrophin-3; PAP: Peripheral astrocyte processes; STAT: Signal Transducer and Activator of Transcription; TRAP: Translational ribosome affinity purification; TSP: Thrombospondin; VGluT1: Vesicular glutamate transporter 1.

## Competing interests

The authors declare that they have no competing interests.

## Authors’ contributions

YY and HH contributed to the writing of the manuscript; HH and LM performed imaging and western experiments. All authors read and approved the final manuscript.
